# Online volunteer laboratories for human subjects research

**DOI:** 10.1371/journal.pone.0221676

**Published:** 2019-08-28

**Authors:** Austin M. Strange, Ryan D. Enos, Mark Hill, Amy Lakeman

**Affiliations:** Institute for Quantitative Social Science and Department of Government, Harvard University, Cambridge, MA, United States of America; Georgia State University, UNITED STATES

## Abstract

Once a fixture of research in the social and behavioral sciences, volunteer subjects are now only rarely used in human subjects research. Yet volunteers are a potentially valuable resource, especially for research conducted online. We argue that online volunteer laboratories are able to produce high-quality data comparable to that from other online pools. The scalability of volunteer labs means that they can produce large volumes of high-quality data for multiple researchers, while imposing little or no financial burden. Using a range of original tests, we show that volunteer and paid respondents have different motivations for participating in research, but have similar descriptive compositions. Furthermore, volunteer samples are able to replicate classic and contemporary social science findings, and produce high levels of overall response quality comparable to paid subjects. Our results suggest that online volunteer labs represent a potentially significant untapped source of human subjects data.

## Introduction

Human subjects research in the social and behavioral sciences has undergone a major transformation in recent decades. Enabled by the Internet, large samples of paid subjects, including nationally-representative surveys, now dominate human subjects research. In particular, the use of low-cost convenience samples such as Amazon’s Mechanical Turk (MTurk) has proliferated across economics, political science, psychology, and other disciplines [1–7] and the availability of these platforms has expanded beyond the English-speaking world, to large and growing academic communities, such as China [8]. These platforms have dramatically reduced the costs of obtaining subjects, allowing for large samples to be recruited rapidly [9]. While a researcher once had to serially collect responses in a laboratory, limited by the physical space available and the size of the local subject pool, a researcher can now collect responses in parallel from virtually anywhere on the globe. Despite this shift, a crucial aspect of human subjects research remains unchanged: for their participation, subjects are rewarded with money, course credit, or other forms of extrinsic motivation.

Is material compensation the only way to attract high-quality subjects? We present the case for a complementary resource that allows for the collection of inexpensive, large, and diverse populations online: the volunteer. We introduce the concept of a scalable, shared online volunteer laboratory and we extensively test the properties of online volunteer subjects from one of these laboratories. We demonstrate that volunteer subjects can generate similar results to those obtained from paid representative and convenience samples. Using the Internet to gather sustainable pools of volunteer subjects at little cost represents a potential major innovation: it also offers numerous benefits to researchers across a range of disciplines by reducing costs, mitigating potential ethical concerns, and leveraging subjects’ intrinsic motivations to avoid potential response quality pitfalls. Moreover, we argue that volunteer subjects may have properties that make them superior to paid subjects, such as greater attention and less incentive to misrepresent themselves. Furthermore, because online volunteer labs, unlike paid platforms, give researchers the ability to recruit and maintain their own sample, these labs can provide control and flexibility not found on other platforms and can potentially become cross-institutional public goods, supporting a broad research agenda.

Volunteer subjects were once a fixture of quantitative human subjects research across social and behavioral sciences [10]. While early designs to leverage the Internet as a research labor-pool noted the potential of volunteer workers [11], online and offline volunteers now only rarely appear in published research. In contrast, paid online samples have proliferated rapidly as sources of survey data.

A significant potential difference between volunteer and paid subjects is their motivation for participating in research. Previous research has demonstrated that volunteer subjects, compared to paid subjects, are on average more motivated by intrinsic motivations [12]—that is a willingness to perform an activity that stems directly from the activity itself, or the “inherent satisfactions” it provides, rather than expectation of reward [13].

Drawing subjects from a pool of intrinsically-motivated volunteers allows a range of potential benefits to accrue to researchers when compared to the use of paid subject pools. Most obviously, volunteer subjects significantly reduce monetary costs to individual researchers. As a benchmark, if a researcher uses MTurk to recruit 1,000 subjects at the U.S. Federal minimum wage of $7.25 per hour for a 10 minute study, the resulting cost would be over $1,200 for subjects alone, plus a 40% surcharge based on the standard MTurk fee of 20% and an additional 20% for tasks with more than 10 workers (see https://requester.mturk.com/pricing). On another paid platform, Lucid, a 10-minute quota-sampled survey can cost approximately $1 per subject [14]. Such costs can be significant for many researchers, especially in the social and behavioral sciences. Moreover, pressure to reduce research costs may contribute to low wages for these subjects, raising ethical concerns [15]. A recent investigation into the salaries of people performing a high volume of tasks on MTurk suggests that average salaries may be as low as $2 per hour [16], far below the federal minimum wage.

Volunteer subjects’ intrinsic motivations for participating also have the potential to improve data quality by reducing the presence of some common pitfalls associated with paid respondents. When subjects are working at an hourly rate (sometimes as a major source of income [15]), they may have an incentive to rush through studies or provide fictitious information, potentially leading to low-quality data. Moreover, individuals sometimes misrepresent their true identity in order to qualify for MTurk tasks that offer higher returns [17]. Recent evidence also suggests that significant amounts of paid online response data may be produced by automated bots [18]. A related concern is that subjects participate multiple times in the same or related experiments [19, 20]. While the severity and implications of these behaviors remains an area of active research, because volunteers are not motivated by an hourly wage, such concerns are minimal: researchers have greater control over the distribution of individual studies to volunteers, and volunteers have little or no incentive to repeat a single study. [18, 21–24].

Of course, all subject pools have advantages and disadvantages. Paid online samples offer the tremendous advantage over offline samples of quickly providing relatively inexpensive subjects to researchers. However, the experiences of several online volunteer laboratories across disciplines and institutions have demonstrated that online volunteer pools can fill a similar role. These online volunteer labs draw on similar technologies to paid online subject pools but leverage the non-monetary motivation of subjects and have shown to be sustainable and scalable [25–27]. Successful examples of online volunteer labs that serve a broad research agenda include Lab in the Wild (https://www.labinthewild.org/) in Computer Science, Volunteer Science (https://volunteerscience.com/) from a consortium of social, behavioral, and computational sciences, and a pan-social science laboratory: the Harvard Digital Lab for the Social Sciences (DLABSS) (http://dlabss.harvard.edu/), from which we will draw data below. Volunteer data collected online has also been successfully used for specific research projects, such as Project Implicit (https://implicit.harvard.edu/implicit/) and the Moral Foundations project (https://moralfoundations.org).

As one example of these labs’ potential, DLABSS has built a standing pool of over 16,000 volunteers and has shown the ability to produce large samples for a range of social science studies. In 2017 and 2018, a typical study reached 403 subjects in 30 days, with a final sample of over 700 subjects. The costs of DLABSS are minimal and there is good reason to believe the model can be extended to other institutions: the essential features of the laboratory are simply a website, Qualtrics survey software, and an email list; volunteers are primarily recruited using social media and other free sources. DLABSS’s substantial volunteer pool has been built in just over three years using a few hours of student labor per week. At many institutions, non-monetary resources such as course credit could also be used to mitigate lab management and maintenance costs.

The potential scalability of online volunteer laboratories is bolstered by the possibility for cross-institutional collaboration. Consistent with models used in survey research (e.g., the Cooperative Congressional Election Study), collaboration across institutions could keep material costs low and aid in sharing non-material resources, such as reputation-driven access to volunteer subjects. Given the flexible and minimal infrastructure of digital volunteer labs, logistical costs to cross-institutional collaboration are potentially quite low.

Of course, despite the advantages of volunteer subjects, labs that rely on intrinsic motivation also have potential disadvantages: though volunteer labs have demonstrated the potential for large, readily available samples of volunteers, these subjects may be acutely unrepresentative, raising external validity concerns. For example, volunteers may be more motivated by academic interest than the general public, or their high levels of intrinsic motivation may be associated with unrepresentative attitudes and behaviors [28]. Given that studies from volunteer pools are already appearing in prominent social science journals (e.g., [29–31]), these possibilities should be investigated.

As such, across four studies we examine the properties of online volunteers and benchmark our analyses through parallel studies of paid subjects. We first compare the motivations of volunteer and paid subjects to test whether volunteer online subjects actually participate because of intrinsic motivation. We then explore potential consequences of this differential motivation by examining the demographic properties of volunteer subjects, the ability to replicate existing studies using volunteers subjects, and the relative quality of volunteer subject responses. All tests reported in this manuscript involving human subjects were approved by Harvard’s Committee on the Use of Human Subjects (CUHS). Consent from both volunteer and paid subjects was obtained electronically.

## Study 1: Motivation of volunteer and paid subjects

Do online volunteer subjects have different motivations than paid subjects from online pools such as MTurk, thus raising the possibility that volunteer subjects may have higher response quality than paid subjects? To investigate this question, we fielded a survey of volunteer and paid subjects.

### Materials and methods

We surveyed the motivations of 742 volunteers on DLABSS and 649 MTurk paid subjects. DLABSS subjects were drawn from the standing pool of DLABSS subjects and MTurk workers were recruited with an advertisement for a “Short survey about Mechanical Turk” and paid $0.50. Eligibility was limited to workers in the United States with a 95% or greater approval rating. We report on the demographic properties of DLABSS volunteers and MTurk subjects in the next study.

Volunteers and paid respondents were tasked with ranking, from least to most important on a 7 point scale, motivations for their own participation in online surveys. The motivations offered were earning money, learning about current affairs, being part of an online community, helping researchers, experiencing studies, passing the time, and helping others. Respondents were also asked how likely they would be to participate in online studies even if there was no chance of compensation, and given the opportunity to describe in detail why they choose to participate in online studies.

### Results

**Fig 1:**
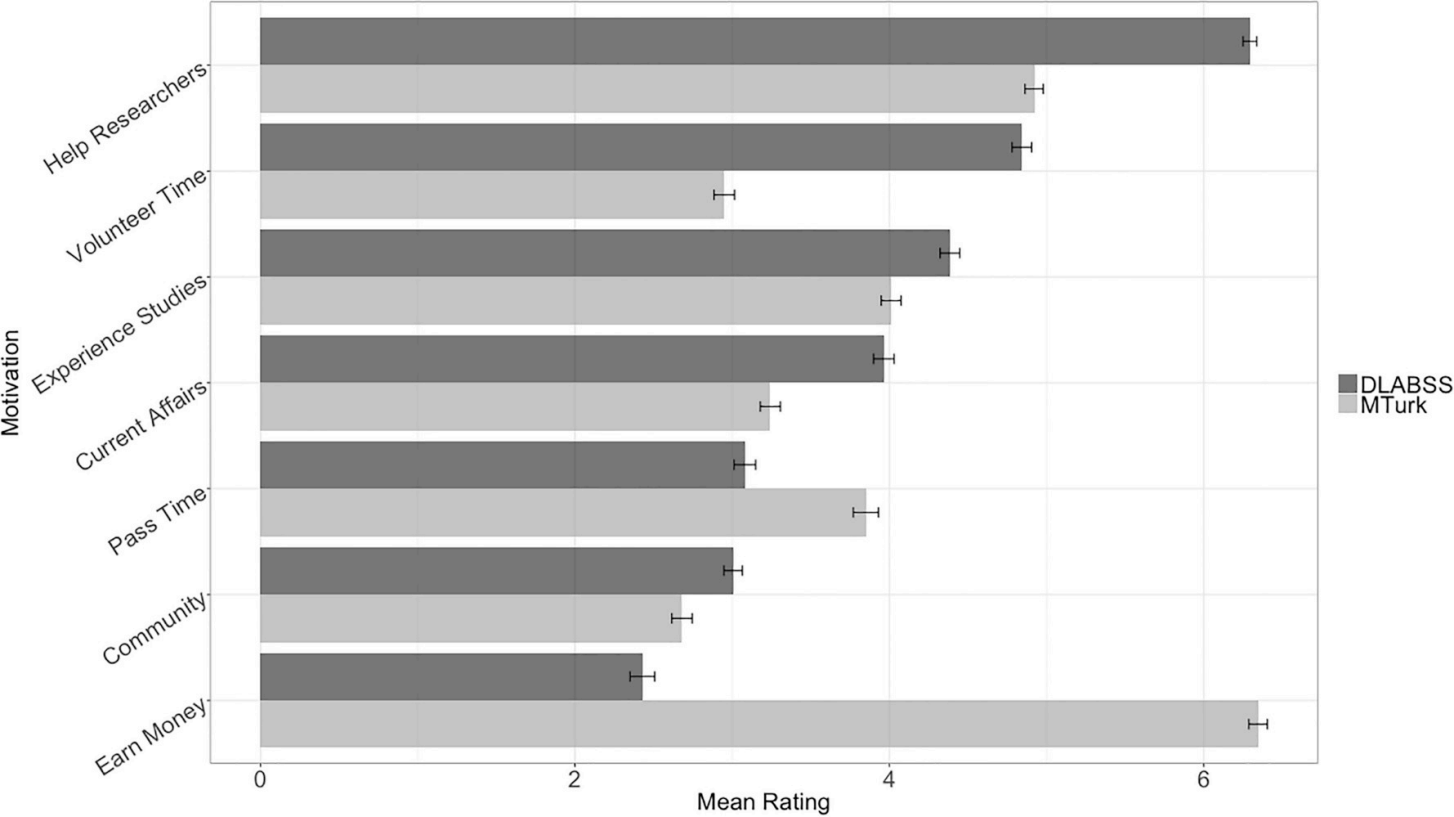
Comparing DLABSS and MTurk self-reported motivations. Black bars represent standard errors of the mean.

Volunteers and paid subjects reported significantly differing motivations for participating in online surveys (Fig 1). Those in the paid subject pool were most motivated by earning money, while volunteers were least motivated by earning money and most motivated by the possibility of helping researchers. Additionally, when asked how likely they would be to participate in such studies even if there was no chance for compensation, only 15.2% of paid subjects said they would be very likely or somewhat likely to do so, while 85.7% of volunteers reported they would be very likely or somewhat likely to continue. When probed to explain their motivations in depth, 43.5% of paid subjects mentioned the word “money”, compared to only 2.6% of volunteers. In short, volunteer and paid online subjects reported fundamentally different motivations for their participation in online surveys, and volunteers reported having substantially higher average levels of intrinsic motivation.

## Study 2: Demographics of volunteer subjects

Though higher average levels of intrinsic motivation of volunteer subjects may increase the propensity for high-quality responses, we may also worry that subjects with the time and inclination to volunteer for studies are acutely unrepresentative. As such, we test the characteristics of volunteer subjects, drawing on the influential study of Berinsky, Huber and Lenz (2012) [3], which is often cited in research using MTurk to justify the validity of that subject population.

### Materials and methods

Following Berinsky, Huber and Lenz (2012), on MTurk we placed an advertisement for a “Survey of Public Affairs and Values” and fielded a similar study on DLABSS for 909 and 705 subjects, respectively. For some questions asked of the entire DLABSS panel and not just this survey, we report the population average for the entire panel. We then compare these populations to the same high-quality representative surveys used by Berinsky, Huber, and Lenz: the online 2008-09 American National Elections Study Panel Study (ANESP), the 2012 American National Elections Study (ANES), and the 2012 Current Population Survey (CPS).

### Results

In Table 1 we report the key demographics of these five samples. In Table B in S2 Appendix, we also compare political behavior and knowledge across these two samples, which was also examined by Berinsky, Huber and Lenz (2012). Generally speaking, volunteer subjects appear similar to respondents from online and offline survey platforms, though important differences do exist. For example, compared to MTurk subjects, volunteers appear more representative of the general population in terms of age. However, compared to the nationally representative samples, volunteers, similar to the paid subjects from MTurk, tend to be more educated and have lower incomes. While MTurk over-represents men, women are over-represented among volunteer subjects. There do not appear to be acute differences between paid and volunteer subjects across demographic characteristics that may be expected to be correlated with intrinsic motivation. For example, compared to MTurk subjects, volunteers are not substantially more educated, wealthy, or white.

**Table 1:** Comparing DLABSS sample demographics to internet and face-to-face samples.

	*Internet sample*			*Face-to-face samples*	
	*DLABSS*	*MTurk*	*ANESP*	*CPS 2012*	*ANES 2012*
Female	57.2% (0.6)	48.0% (1.9)	57.6% (0.9)	51.9% (0.2)	52.0% (0.1)
Education (mean years)	15.1 (0.0)	14.9 (0.1)	16.2 (0.1)	13.4 (0.0)	13.6 (0.1)
Age (mean years)	43.3 (0.2)	37.8 (0.5)	49.7 (0.3)	46.7 (0.1)	47.3 (0.4)
Mean income	$48,203 ($480)	$43,592 ($1,168)	$69,043 ($749)	$61,977 ($138)	$63,199 ($1,274)
Median income	$37,500	$37,500	$67,500	$55,000	$32,500
Race					
White	74.4 (0.5)	78.3 (1.6)	83.0 (0.7)	79.5 (0.1)	74.5 (1.0)
Black	6.3 (0.3)	8.4 (1.0)	8.9 (0.7)	12.2 (0.1)	12.2 (0.7)
Hispanic	7.9 (0.3)	7.7 (1.0)	5.0 (0.4)	15.0 (0.1)	10.9 (0.7)
Marital Status					
Married	42.5 (1.5)	42.7 (1.0)	56.8 (0.9)	53.8 (0.2)	53.2 (1.1)
Housing status					
Own home	51.8 (1.5)	49.5 (1.9)	80.8 (0.8)		71.5 (1.0)
Religion					
None	43.9 (1.5)	40.0 (1.8)	13.1 (0.8)		21.3 (0.9)
Protestant	20.0 (1.2)	25.4 (1.6)	38.7 (1.4)		33.3 (1.1)
Catholic	16.8 (1.1)	20.4 (1.5)	22.9 (1.0)		22.7 (0.9)
Region of the US					
Northeast	22.8 (0.5)	21.5 (1.6)	16.9 (0.7)	18.2 (0.1)	18.2 (0.8)
Midwest	21.0 (0.5)	25.4 (1.7)	28.3 (0.9)	21.6 (0.1)	22.6 (0.9)
South	31.1 (0.6)	38.2 (1.9)	31.4 (0.9)	37.0 (0.2)	37.2 (1.1)
West	25.2 (0.6)	14.9 (1.4)	23.4 (0.8)	23.2 (0.1)	22.1 (0.9)
Party Identification					
Democrat	47.9 (0.6)	44.3 (1.9)			
Independent/Other	30.3 (0.5)	30.1 (1.7)			
Republican	21.8 (0.5)	22.8 (1.6)			
Ideology					
Liberal	59.9 (0.5)	62.6 (1.9)			
Conservative	32.9 (0.5)	37.4 (1.9)			
Registration/turnout					
Registered	89.3 (0.9)	91.6 (1.0)	92.0 (0.7)	71.2 (0.1)	72.8 (1.0)
Voted in 2008	80.1 (1.4)	73.8 (1.7)	89.8 (0.5)	61.8* (0.2)	70.2* (1.0)
N	909-8,122	673-705	2,727-3,003	92,311-102,011	2,004-2,054

Standard errors are in parentheses. N is a range because of differing missingness across survey questions.

* indicates turnout in 2012. Political interest is on a 5-point scale with 5 indicating high interest.

### Discussion

While not entirely representative of the general population, online volunteer subjects are not acutely less representative than online paid subjects. Notably, online volunteer laboratories can also shape their own subject pools, allowing them to seek certain demographics by advertising through certain channels and, thereby, increase the representativeness of the sample over time.

## Study 3: Replication of existing studies using volunteer subjects

Having established that volunteer samples can look broadly similar to nationally representative and MTurk samples, we turn to the question of whether the results of well-established studies, originally done using other samples, can be replicated with volunteer subjects.

### Materials and methods

We report the replication of six classic and contemporary social and behavioral science experiments, starting with three experiments that have been used to test paid online samples [3] and moving to three other experiments in order to further test the potential of volunteer samples. The latter three studies arguably involve more complex sociopolitical attitudes than the first three.

All six of the studies were originally conducted on other populations, including the General Social Survey (GSS), Knowledge Networks (KN), and a group of students. Three studies were subsequently replicated by Berinsky, Huber and Lenz (2012) on MTurk to test the properties of paid subjects.

We replicated all six of the studies using volunteer subjects on DLABSS and for two of the studies that had not previously been replicated on MTurk, we also conducted a replication on MTurk. With these replications, we can test whether DLABSS can also replicate the studies whose replication is used to justify the use of paid online samples. In addition, we can test whether DLABSS can replicate other prominent social and behavioral science findings.

In judging whether a replication was successful, we considered three criteria: First, the coefficient on the treatment variable must have the same directionality as in the original study. Second, in line with the original six studies we replicate, we consider a coefficient to be statistically significant if the associated *p* < .05. Third, the treatment coefficient must be of the same approximate magnitude as the original study, so that rough effect sizes across the original and replication studies are comparable.

We began by replicating with volunteer subjects the three experiments previously replicated using MTurk by Berinsky, Huber and Lenz (2012) [3]. These were: First, Rasinski 1989 [32] (Table A in S3 Appendix), who using data from the GSS found that framing policy choices dramatically changes stated preferences for redistributive policies, so that citizens are much more likely to support redistribution when it is worded as “assistance to the poor” compared to “welfare.” Second, the well-known Asian Disease Problem popularized by Tversky and Kahneman (1981) [33] (Table B in S3 Appendix), who found that framing policy options in terms of losses (deaths) rather than gains (lives saved) leads to stronger preferences for probabilistic outcomes. Third, Kam and Simas (2010) [34] (Table C in S3 Appendix), who demonstrate that individuals willing to accept higher amounts of risk are more likely to support probabilistic policy outcomes.

We then moved to three additional replications on DLABSS and MTurk. First, we replicated Tomz (2007) [35]’s study on the microfoundations of audience costs in international relations (Table D in S3 Appendix), demonstrating that citizens are more likely to disapprove of their head of state when he or she makes an empty threat versus not making a threat at all. Second, Hainmueller and Hiscox (2010) [36]’s experimental study that challenges traditional, egoistic political economy theories about preferences for immigration (Fig A in S3 Appendix). Their results suggest citizens universally prefer high-skilled immigrants regardless of their own income status, education level and other covariates, lending support to theories of sociotropic preferences for immigration. Third, we replicated Gadarian and Albertson (2014) [37]’s finding that different levels of anxiety affect how individuals search for information about immigration.

### Results

Each of the six replications described above had the same approximate magnitude, directionality, and level of statistical significance as in the original studies. Table 2 summarizes these replications, with additional descriptions in S3 Appendix.

**Table 2:** Experimental social science replicated with volunteer subjects on DLABSS.

Replicated Study	Dependent Variable	N	MTurk	N	Original	N
Tversky and Kahneman (1981)	Risk acceptance	539	✔	450	students	307
Rasinski (1989)	Support for gov’t spending	788	✔	329	GSS	1,470
Kam and Simas (2010)	Policy acceptance	752	✔	699	KN	752
Tomz (2007)	Audience costs	495			KN	1,127
Hainmueller and Hiscox (2010)	Immigration attitudes	736	✔*	833	KN	1,601
Gadarian and Albertson (2014)	Information seeking	668	✔*	736	KN	384

The first three studies were replicated by Berinsky, Huber and Lenz (2012) using paid MTurk subjects. “Original” column indicates the original sample, of which we are aware, other than MTurk or DLABSS, on which the study was carried out and is not an exhaustive list of replications. The first N column is the number of subjects on DLABSS, the second N is the number of subjects on MTurk, and the third N is the number of subjects on the original platform by the original researchers. A * next to the ✔ for MTurk indicates that we carried out the MTurk replication ourselves.

### Discussion

The replication of six studies, across a range of topics and with the original studies using a variety of platforms, gives us confidence in the ability of volunteer subjects to provide valid subjects for the social and behavioral sciences.

In addition to these replications, many researchers have replicated studies in the course of performing research on DLABSS. We collected information on replications by surveying researchers who had used DLABSS, then communicating with researchers who indicated that they used DLABSS volunteers to replicate studies conducted on other platforms. In doing so, we identified 10 additional replications that other researchers have conducted using volunteer subjects. We give more information on these studies in Table E in S3 Appendix. To the best of our knowledge, there are no unreported failures to replicate using DLABSS, but we cannot rule out this possibility.

## Study 4: Response quality of volunteer compared to paid subjects

Having established that volunteer subjects have different motivations than paid subjects, but are nonetheless broadly demographically similar and can be used to replicate a range of studies, we now turn to direct tests of the response quality of volunteer subjects.

### Materials and methods

In order to establish that any observed differences in response quality are not idiosyncratic to subject matter, we used two different surveys, each fielded on both DLABSS and MTurk. Due to missingness on some covariates and attrition, effective sample sizes vary across the tests of response quality we present below (see Table A in S4 Appendix for details). The two surveys vary only in substantive issue area: the first focused on religion and secularism in the United States (N = 557 DLABSS, 459 MTurk), and the second on foreign economic policies (N = 519 DLABSS, 482 Mturk). In Table B in S4 Appendix we compare the demographic composition of the volunteer and paid samples and find balance across a number of covariates. Each survey took about 15 minutes to complete. DLABSS subjects were invited to take part in one of the two surveys, and MTurk subjects were invited into a single task and then randomized into one of the two surveys. MTurk subjects were recruited by an advertisement to “Share your thoughts on pressing political issues!” and were compensated $1 for their time. Eligibility was limited to workers in the United States with a 95% or greater approval rating. DLABSS subjects could come from anywhere in the world, but we remove the 144 subjects from outside the United States in the analyses.

As there are a number of approaches to measuring response quality, we adopt eleven tests across seven quality dimensions in order to avoid over-reliance on any specific measure. Drawing on relevant literature [38–40], we measure response quality based on subjects’ propensity to 1) invest time in reading a prompt and answering questions; 2) answer grid-style questions without engaging in straightlining; 3) invest effort into open-ended responses; 4) offer committal answers; 5) answer opinion questions consistently at different points in the survey; 6) avoid skipping questions; and 7) catch an embedded attention check. Table C in S4 Appendix summarizes the design of each test. S4 Appendix further describes our measurement strategies and presents detailed results for each test.

We note that some of these measures, such as straightlining over a large set of responses, are clear measures of response quality, but others are more ambiguous. For example, a respondent who skips a question may do so in order to complete the survey more rapidly or to avoid expending additional effort (low quality), or may skip the question due to uncertainty about the correct answer or ambiguity about her own feelings on the topic (conceivably high quality).

### Results

**Fig 2:**
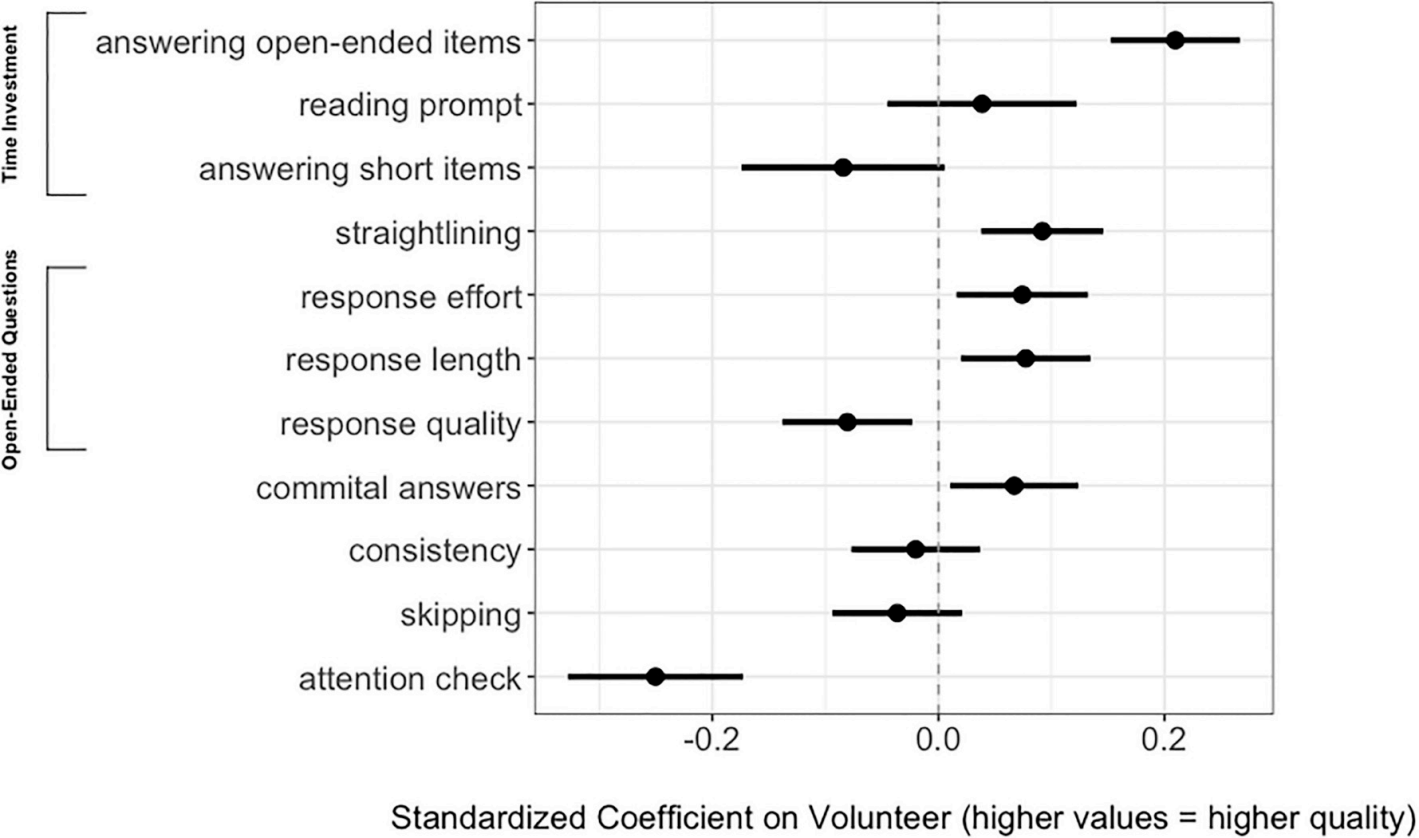
Standardized “Volunteer” coefficients for all response quality tests. Coefficients on “reading prompt”, “answering short items”, and “attention check” represent results for a single substantive survey, because they were not included in both questionnaires.

Fig 2 presents a summary of the results for each test across both surveys. Our quantity of interest is the average difference in response quality between volunteer and paid subjects. We measure this difference from an OLS regression of each dependent variable on a dummy variable for whether the subject was a volunteer or not, with positive values representing higher quality. The coefficient on volunteer is thus the added quality of a volunteer subject. We control for individual demographic covariates that have some imbalance across the samples, including age, income, education, race, frequency of religious service attendance, religious tradition, political ideology, party identification, and a dummy for the survey topic (See S4 Appendix). Fig A in S4 Appendix includes disaggregated multivariate results for the secularism and foreign economic policy surveys. Fig B in S4 Appendix includes bivariate results.

Volunteers appear to have response quality similar to, and in some cases better than, paid subjects. On five tests of quality, including time invested reading a prompt, propensity for straightlining, open-ended response length, and committal answers, volunteers produced statistically significantly higher-quality responses (p<.05). On four other tests, volunteers and paid subjects were statistically indistinguishable (p>.05). In two tests, paid subjects’ responses were of higher quality (p<.05): first, open-ended responses, for which paid subjects scored higher on a subjective measure of overall quality. Second, paid subjects were more likely to correctly address the attention check, or “screener,” embedded in a text block in the secularism survey.

### Discussion

Paid subjects outperforming volunteers on attention to the screener question may indicate that paid subjects were more carefully reading the survey, although some of the difference may have come from familiarity with these checks due to the frequency with which paid subjects participate in studies. The fact that volunteers spent significantly more time than paid subjects reading the text block in which the attention check was embedded, even after controlling for background covariates, supports this interpretation, but further research is needed.

We also note a potential response quality difference between volunteer and paid subjects that is not directly tested here and also deserves further investigation: subject attrition, which may cause bias in experimental research if the attrition is differential across treatments. Zhou et al (2016) [41] replicate existing MTurk studies and find that every one had an attrition rate of greater than 20%, sometimes exceeding 50%. In contrast, selective attrition may be less severe in volunteer labs, perhaps due to the intrinsic motivation of subjects: the mean and median attrition rate for studies in DLABSS are 22.5% and 20%, respectively (these numbers decrease considerably for surveys under 10 minutes in length; see Fig D in S4 Appendix).

## Conclusion

Building on the innovation of online crowd-sourcing, we argue that volunteer laboratories can similarly extend the accessibility and quality frontiers of human subjects research. A large body of literature has demonstrated the viability of paid online subjects for social and behavioral science research [1, 3, 42]. Volunteer subjects resembling these paid populations add another source of data for human subjects researchers. To demonstrate this potential, we compared the sample properties of an online volunteer laboratory to those of several widely used paid subject pools. We then replicated a diverse range of experimental studies using a volunteer laboratory, and explored the relative quality of data produced by volunteer subjects through a series of tests.

Of course, the tests of volunteer subjects were all conducted on a single volunteer laboratory. While the presence of other online volunteer laboratories has demonstrated that such laboratories can be successful across a range of subjects and institutional settings, testing the properties of other labs will be important for understanding whether the quality and demographic makeup of subjects are idiosyncratic to the particular lab on which we conducted these tests.

The quality and scalability of online volunteer pools is encouraging. Of course, volunteer laboratories, like all data sources, have advantages and disadvantages that will allow them to occupy a particular niche in human subjects research, and will likely emerge as complements to other survey platforms. Coordination across institutions would significantly increase the efficiency of volunteer laboratories, in much the same way that economies of scale in designs like the Cooperative Congressional Election Study have greatly improved the efficiency of survey research. We urge researchers to undertake such collaborations.

## Supporting information

S1 Appendix Appendix for *DLABSS Operations*. This appendix provides additional information on the operations and properties of the volunteer lab used in this paper, the Harvard Digital Lab for the Social Sciences (DLABSS).
(PDF)

S2 Appendix Appendix for *Supplemental Materials for Study 2*. This appendix includes additional information on the attributes of the volunteer subjects used throughout this manuscript.
(PDF)

S3 Appendix Appendix for *Supplemental Materials for Study 3*. In this appendix, we offer additional information regarding replications conducted on DLABSS.
(PDF)

S4 Appendix Appendix for *Supplemental Materials for Study 4*. This appendix includes additional information on the design and results of individual response quality tests for volunteer and paid subjects.
(PDF)
