# Supplementary material for: Online volunteer laboratories for human subjects research
Source: PLoS One. 2019 Aug 28;14(8):e0221676. doi: 10.1371/journal.pone.0221676 (PMC6713332; doi:10.1371/journal.pone.0221676)
Supplement: S1 Appendix — This appendix provides additional information on the operations and properties of the volunteer lab used in this paper, the Harvard Digital Lab for the Social Sciences (DLABSS). (PDF) [file pone.0221676.s001.pdf]

Appendices for *Online Volunteer Laboratories for Human Subjects Research*

S1 Appendix: DLABSS Operations

This appendix provides additional information on the operations and properties of the volunteer lab used in this paper, the Harvard Digital Lab for the Social Sciences (DLABSS). DLABSS was created in 2014 with the principle that volunteer online subjects should be a public good to researchers with diverse substantive interests. DLABSS is staffed by several part-time researchers, including a faculty member and graduate and undergraduate students. The basic structure is a website that serves as a clearinghouse for experimental and other survey-based behavioral and social science studies. The research instruments are not directly hosted on DLABSS, rather staff curate links to the instruments along with basic descriptions on the website. This simple structure allows researchers to host their instruments on any other convenient platform, such as Qualtrics or an application built specifically for the research. Using Qualtrics, the lab collects basic demographic and attitudinal data on all subjects in the lab. This simple structure of the lab allows researchers to collect response data for virtually any type of experimental, survey-based research question.

Subjects are recruited using a variety of methods, including organic web search, paid search, social media, and email. Thus far, Craigslist, Reddit, social media, and targeted ad campaigns on email newsletters for specific demographic groups have been the primary channels for recruiting new subjects.

Specific studies are often used to attract subjects and, as such, subjects for a given study usually consist of two types: 1) new subjects that enter an experiment after being recruited from the web and 2) subjects from the existing subject pool who enter an experiment after being directly solicited, usually by email. DLABSS staff email a fraction of the active subject pool each week to invite them to participate in the latest studies. Subjects are emailed biweekly on average. These email solicitations can allow a researcher to shape her subject pool, if desired, by targeting certain populations based on known covariates. In this manner, DLABSS shares this targeting ability with other opt-in internet panels, but maintains the low-cost structure of crowd-sourced research. Figures B and A depict the

Figure A: United States Distribution of DLABSS Volunteer Subjects

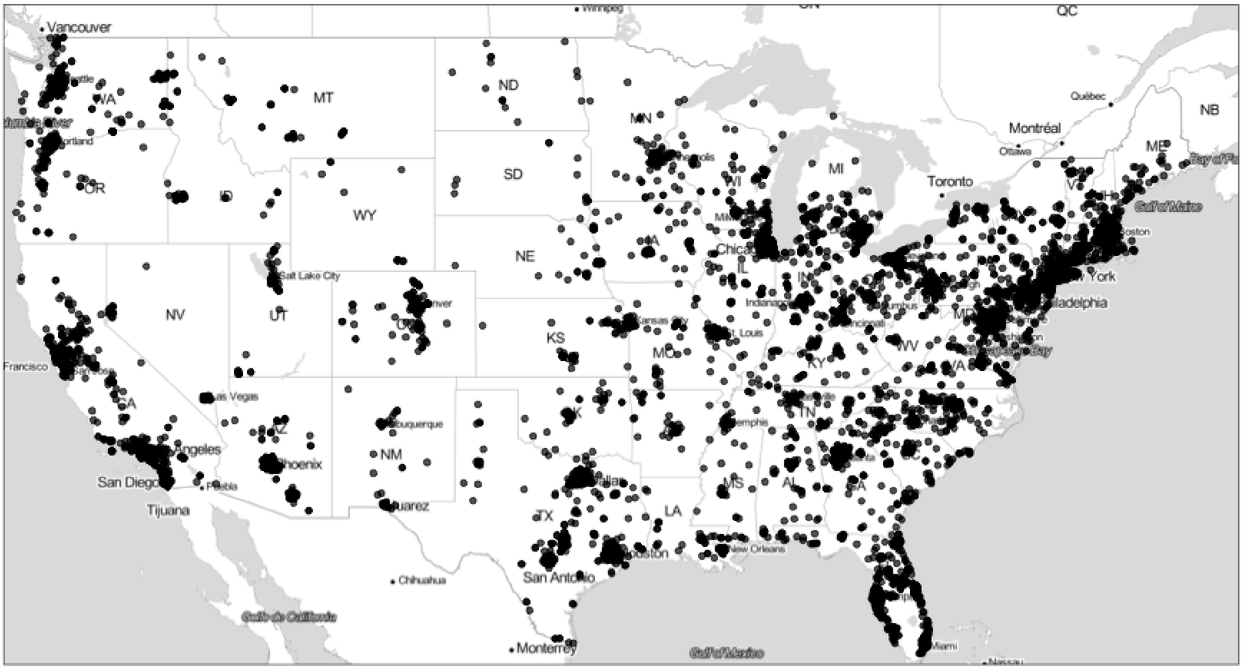

A total of 10,506 points are plotted on this map, each one representing a single DLABSS participant.

geographic locations of DLABSS volunteers

Since its inception, DLABSS has hosted over 100 studies using human subjects. 35% of these were Harvard faculty projects, 60% were graduate student projects, and 5% were undergraduate student projects. As of July 2019, the active DLABSS volunteer pool includes over 16,500 subjects. This means that since its inception, DLABSS has attracted almost 10 new subjects per day. Notably, these descriptive figures include weekends and summer and other academic holidays, and subject recruitment is significantly higher during the periods of the academic calendar where DLABSS staff are working. Subject acquisition discussed above is linked to the nature and diversity of research studies hosted on DLABSS.

Prior to beginning operation, DLABSS secured approval from an institutional review board to recruit subjects and collect demographic data. Researchers using the lab secure human subjects approval for their individual studies. The use of deception in studies is left to the discretion of the researcher. Studies have come from a variety of disciplines, including

Figure B: World-wide Distribution of DLABSS Volunteer Subjects

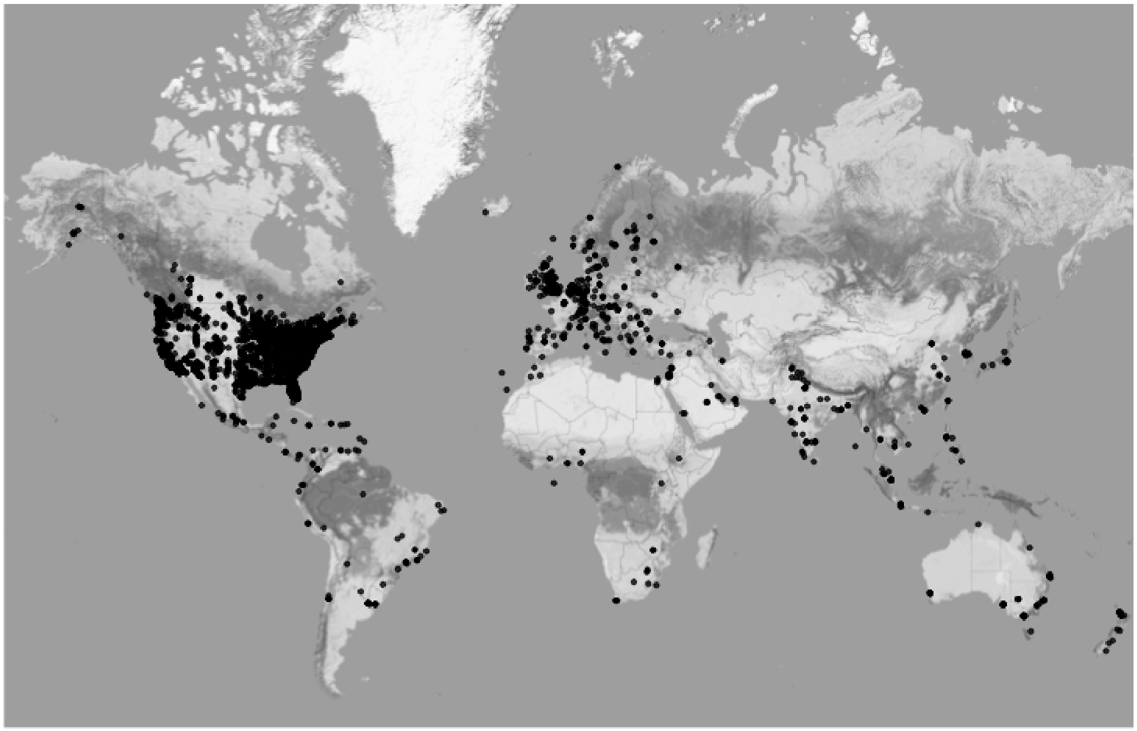

A total of 11,717 points are plotted on this map, each one representing a single DLABSS participant.

business, economics, sociology, political science, public policy, and psychology. The majority of studies on DLABSS have been collaborative and have involved two or more researchers.
