# Supplementary material for: Online volunteer laboratories for human subjects research
Source: PLoS One. 2019 Aug 28;14(8):e0221676. doi: 10.1371/journal.pone.0221676 (PMC6713332; doi:10.1371/journal.pone.0221676)
Supplement: S2 Appendix — This appendix includes additional information on the attributes of the volunteer subjects used throughout this manuscript. (PDF) [file pone.0221676.s002.pdf]

Appendices for *Online Volunteer Laboratories for Human Subjects Research*

S2 Appendix: Supplemental Materials for Study 2

This appendix includes additional information on the attributes of the volunteer subjects used throughout this manuscript. It provides demographic properties of these volunteer subjects and additional information with respect to their political knowledge and preferences.

Table A compares the DLABSS volunteer pool with samples from online convenience samples used by Berinsky, Huber, and Lenz (2012). Tables B and C compare the political knowledge and policy attitudes, respectively, of paid and online subjects.

Volunteers have political knowledge largely similar to paid MTurk subjects and slightly higher than the representative ANESP sample. Vounteers report relatively similar policy attitudes to other survey samples in Table C. The exceptions to this are immigration and gay marriage attitudes, on which the DLABSS sample reports more liberal attitudes than some other platforms. However, on immigration DLABSS subjects report attitudes similar to those of a face-to-face sample from the ANES in 2012. On gay marriage, DLABSS subjects report attitudes relatively similar to MTurk subjects, who are also more liberal in their attitudes than nationally representative samples.

Table A: Comparing Subject Properties across DLABSS and Other Convenience Samples

			Convenience samples			
					Adult samples (Berinsky and Kinder 2006)	
Demographics	*DLABSS*	*MTurk*	*Student samples (Kam et al. 2007)*	*Adult sample (Kam et al 2007)*	*Experiment 1: Ann Arbor, MI*	*Experiment 2: Princeton, NJ*
Female	55.6% (0.7)	48.0% (1.9)	56.7% (1.3)	75.7% (4.1)	66.0%	57.1 %
Age (mean years)	44.1 (0.2)	37.8 (0.5)	20.3 (8.2)	45.5 (.916)	42.5	45.3
Education (mean years)	15.2 (0.0)	14.9 (0.1)	–	5.48 (1.29)	15.1	14.9
White	74.7 (0.5)	78.3 (1.6)	42.5	82.2 (3.7)	81.4	72.4
Black	6.4 (0.3)	8.4 (1.0)			12.9	22.7
Party identification						
Democrat	46.9 (0.6)	44.3 (1.9)			46.1	46.5
Independent/Other	29.6 (0.6)	30.1 (1.7)			37.6	27.7
Republican	23.5 (0.5)	22.8 (1.6)			16.3	25.8
N	807-6,280	673-705	277-1428	109	141	163

DLABSS and MTurk results from December 2016, all other results from Berinsky, Huber, and Lenz (2012). Note that the Education in the Kam et al 2007 sample is a ordinal indicator, rather than years, and is as reported in Berinsky, Huber, and Lenz (2012).

Table B: Comparing DLABSS sample demographics to internet and face-to-face samples- Political Behavior and Knowledge Attributes

		Internet sample		*Face-to-face sample*
	DLABSS	*MTurk*	*ANESP*	*ANES 2012*
Political Interest	3.84 (0.03)	3.62 (0.04)	2.71 (0.02)	3.34 (0.03)
Political knowledge (% correct)				
Presidential succession after Vice President	67.7 (1.4)	60.3 (1.9)	65.2 (2.0)	
House vote percentage needed to override a veto	75.8 (1.3)	87.9 (1.2)	73.6 (1.3)	
Number of terms to which an individual can be elected president	89.4 (0.9)	97.4 (0.6)	92.8 (0.7)	
Length of a U.S. Senate term	51.8 (1.5)	62.5 (1.8)	37.5 (1.3)	
Number of Senators per state	78.5 (1.2)	83.2 (1.4)	73.2 (1.2)	
Length of a U.S. House term	51.3 (1.5)	49.3 (1.9)	38.9 (1.3)	
Average	69.1	73.5	63.5	
N	909 - 8,122	673 - 705	2,727 - 3,003	2,004 - 2,054

Standard errors are in parentheses. N is a range because of differing missingness across survey questions. Political interest is on a 5-point scale with 5 indicating high interest.

Table C: Comparing DLABSS sample policy attitudes

	DLABSS	*MTurk*	*Internet sample ANESP*	*Face-to-face samples ANES 2012*
Favor prescription drug benefit for seniors	75.7% (1.3)	71.6% (1.7)	74.8% (1.1)	
Favor universal health care	54.5 (1.5)	58.8 (1.9)	41.7 (1.2)	
Favor citizenship process for illegals	62.9 (1.5)	48.1 (1.9)	42.7 (1.2)	63.7 (1.1)
Favor a constitutional amendment banning gay marriage	10.2 (0.9)	21.3 (1.5)	55.4 (1.2)	57.1 (1.1)*
Favor raising taxes on people making more than $200,000	69.9 (1.4)	67.2 (1.8)	55.4 (1.2)	79.3 (0.9)**
Favor raising taxes on people making less than $200,000	7.5 (0.8)	8.9 (0.1)	7.1 (0.6)	
N	1,041-1,044	703-705	1,614-1,618	1,995-2,026

* *Gay and lesbian couples should be allowed to form civil unions but not legally marry, or there should be no legal recognition of a gay or lesbian couple's relationship*
** *Increasing income taxes on people making over one million dollars per year.*

References

Berinsky, Adam J, Gregory A Huber, and Gabriel S Lenz. 2012. "Evaluating online labor markets for experimental research: Amazon. com's Mechanical Turk." *Political Analysis* 20(3): 351–368.
