# Supplementary material for: Online volunteer laboratories for human subjects research
Source: PLoS One. 2019 Aug 28;14(8):e0221676. doi: 10.1371/journal.pone.0221676 (PMC6713332; doi:10.1371/journal.pone.0221676)
Supplement: S3 Appendix — In this appendix, we offer additional information regarding replications conducted on DLABSS. (PDF) [file pone.0221676.s003.pdf]

Appendices for *Online Volunteer Laboratories for Human Subjects Research*

S3 Appendix: Supplemental Materials for Study 3

In this appendix, we offer additional information regarding replications conducted on DLABSS.

A Core Replications: 6 Replications Conducted on DLABSS Explicitly for this Study

In Tables A, B, C, and D, and in Figure A we display detailed results for the replications of three well-known studies mentioned in the manuscript.

Table A: Replication of Rasinski (1989) in DLABSS

	Platform	Poor	Welfare	Difference	p	n
1	DLABSS	64	39	25	<.001	788
2	General Social Surveys (GSS)	64	23	37	<.001	1470
3	MTurk (Berinsky et al. 2012)	55	17	38	<.001	329

Cells represent percent of respondents favoring a policy with each frame. P values are from a T-test of difference of means.

Table B: Replication of Tversky and Kahneman (1981) in DLABSS

	Platform	Lives Saved	Lives Lost	Difference	p	n
1	DLABSS	63	34	29	<.001	539
2	MTurk (Berinsky et al. 2012)	74	38	36	<.001	450
3	Tversky and Kahneman 1981	72	22	50	<.001	307

Cells are percent of respondents choosing non-probabilistic (certain) outcome with each frame.

Table C: Replication of Kam and Simas (2010) in DLABSS

	Kam and Simas (2010)			*Berinsky et al. MTurk Replication*			*DLABSS Replication*		
	(H1a) Mortality frame and risk acceptance	*(H1b) Adding controls*	*(H2) Frame x Risk acceptance*	*(H1a)*	*(H1b)*	*(H2)*	*(H1a)*	*(H1b)*	*(H2)*
Mortality frame	1.068	1.082	1.058	1.180	1.180	1.410	1.011	1.026	1.437
in Trial 1	(0.10)	(0.10)	(0.29)	(0.10)	(0.10)	(0.31)	(0.11)	(0.11)	(0.36)
Risk	0.521	0.628	0.507	0.760	0.780	0.990	1.024	1.029	1.424
acceptance	(0.31)	(0.32)	(0.48)	(0.29)	(0.31)	(0.42)	(0.33)	(0.34)	(0.46)
Female		0.105			-0.018			-0.013	
		(0.10)			(0.11)			(0.11)	
Age		0.262			0.110			0.443	
		(0.22)			(0.31)			(0.24)	
Education		-0.214			0.025			-0.056	
		(0.20)			(0.23)			(0.23)	
Income		0.205			-0.024			-0.022	
		(0.23)			(0.23)			(0.21)	
Partisan		0.038			0.006			0.013	
ideology		(0.19)			(0.15)			(0.13)	
Risk acceptance x			0.023			-0.450			-0.827
Mortality frame			(0.62)			(0.58)			(0.66)
Intercept	-0.706	-0.933	-0.700	-1.060	-1.100	-1.190	-1.098	-1.256	-1.309
	(0.155)	(0.259)	(0.227)	(0.170)	(0.290)	(0.230)	(0.187)	(0.257)	(0.255)
N	752	750	752	699	699	699	634	597	634

Cells are signs and p-values for probit regressions of individual-level acceptance of probabilistic policy outcomes on risk acceptance attitudes (top row) and other covariates.

Figure A: Replication of Hainmueller and Hiscox (2010): Support for Highly- and Low-skilled Immigration among DLABSS Respondents

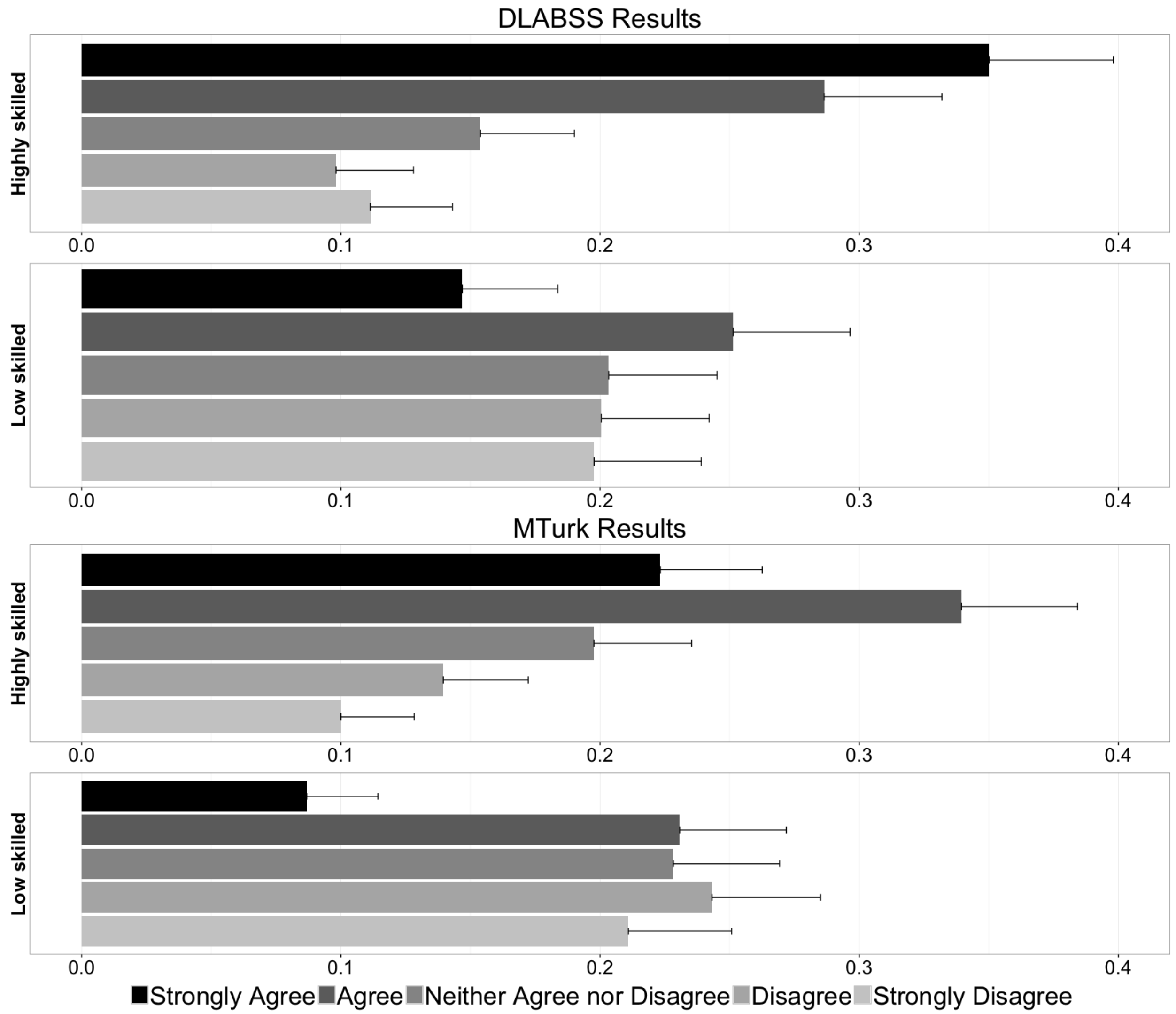

Whiskers are the upper bounds of 95% confidence intervals for proportions. Respondents in the "highly-skilled" group were asked "Do you agree or disagree that the US should allow more highly skilled immigrants from other countries to come and live here? (emphasis added)?" Respondents in the " low-skilled" group were asked "Do you agree or disagree that the US should allow more low-skilled immigrants from other countries to come and live here? (emphasis added)?"

Table D: Replication of Tomz (2007) in DLABSS

	DLABSS Replication of Tomz (2007) Table 1				Tomz (2007 Table 1)			
	Public reaction to empty threat (%)	*Public reaction to staying out (%)*	*Difference in opinion (%)*	*Summary of differences (%)*	*Public reaction to empty threat (%)*	*Public reaction to staying out (%)*	*Difference in opinion (%)*	*Summary of differences (%)*
Disapprove								
Disapprove very strongly	27 (21 to 32)	14 (10 to 19)	12 (6 to 20)	14 (6 to 22)	31 (27 to 35)	20 (17 to 23)	11 (6 to 17)	16 (10 to 22)
Disapprove somewhat	30 (25 to 36)	15 (11 to 20)	15 (8 to 23)		18 (14 to 21)	13 (10 to 16)	5 (0 to 9)	
Neither								
Lean toward disapproving	5 (3 to 9)	14 (10 to 19)	-8 (-14 to -3)	-3 (-12 to 5)	8 (6 to 11)	9 (7 to 11)	0 (-3 to 3)	-4 (-9 to 2)
Don't lean either way	10 (7 to 14)	13 (9 to 17)	-2 (-8 to 4)		21 (17 to 24)	21 (18 to 24)	0 (-5 to 4)	
Lean toward approving	12 (9 to 17)	12 (8 to 16)	0 (-5 to 7)		8 (6 to 11)	11 (9 to 14)	-3 (-6 to 0)	
Approve								
Approve somewhat	10 (6 to 13)	21 (16 to 27)	-12 (-18 to -5)	-9 (-17 to -2)	8 (5 to 10)	13 (11 to 16)	-6 (-9 to -2)	-12 (-17 to -8)
Approve very strongly	5 (3 to 8)	11 (8 to 16)	-6 (-11 to -1)		6 (4 to 9)	13 (10 to 16)	-7 (-10 to -3)	

The table gives the percentage of respondents who expressed each opinion. Bayesian 95 percent credible intervals appear in parentheses.

B Additional Replications: 10 Replications Using DLABSS Reported by Other Researchers

In the manuscript and in the previous section, we present details on 6 experiments we explicitly replicated for this study. In Table E and the text below, we provide information on 10 additional replications reported by researchers while using DLABSS.

Researchers have used DLABSS volunteers to replicate findings across a range of topics (Tversky and Kahneman 1981, Rasinski 1989, Tomz 2007, Hainmueller and Hiscox 2010, Kam and Simas 2010, Gadarian and Albertson 2014, Krosch et al. 2013, Enos and Carney 2017, Enos and Celaya 2018, Mahler 2016, Hankinson 2018, Bonikowski and Zhang 2017, Kaufman 2018, Kaufman, King and Komisarchik Forthcoming, Saha and Weeks 2018, Mozer et al. 2018). We provide additional details here to offer a broader sense of the variety of research volunteer laboratories can reproduce.

Several studies hosted on DLABSS have explored racial politics. One study (Enos 2017), using both DLABSS and Qualtrics' proprietary survey panel, attempted to replicate findings from prominent recent studies that, using small MTurk samples, found significant links between political ideology and visual perceptions of race (Krosch et al. 2013, Krosch and Amodio 2014). Another study tested how spatial segregation affects perceptions of similarity in human faces across DLABSS and MTurk (Enos and Celaya 2018). Several DLABSS studies also investigated the properties of Modern Racism Scales (Sears and Kinder 1971), finding similar distributions of racial attitudes as those in the Cooperative Campaign Analysis Project (CCAP) survey and replicated experimental results on the nationally representative Time Sharing for Experimental Social Science (TESS) panel and MTurk (Enos and Carney 2017).

Another researcher used DLABSS to study populism. DLABSS and MTurk samples produced similar results, while a Qualtrics panel, which was manipulated to be dispropor-

tionately conservative, produced larger effects (Bonikowski and Zhang 2017).

In the context of studying blocked randomization designs, a researcher studied a variant of the Tomz (2007) study referenced above and replicated the results on MTurk and DLABSS (Tomz 2007, Kaufman and Kim 2017). Another team crowdsourced perceptions of the compactness of legislative districts on both MTurk and DLABSS with similar results between the two platforms (Kaufman, King and Komisarchik Forthcoming). Researchers also used MTurk and DLABSS to validate a computational model of sentiment analysis of survey questions with similar results across the platforms (Kaufman 2018).

Finally, while studying the effects of altruistic voting behavior on voting outcomes, a researcher used a representative Danish sample from Epinion and replicated the result on DLABSS with U.S. subjects (Mahler 2016). Another researcher replicated a survey experiment from MTurk on preferences for housing allocation based on the geographic location of the housing (Hankinson 2018).

Table E: Experimental social science replicated on DLABSS: Additional Studies

Replicated Study	Dependent Variable	N	MTurk	N	Other	N
Krosch et al. (2013)	Perceptions of race	204	✓	31	Qualtrics	708
Enos and Carney (2015)	Racism scales	1,478	✓	4,488	TESS	733
Enos and Celaya (2015)	Perceptions of race	365	✓	716		
Mahler (2016)	Voting outcomes	400			Epinion	2,000
Hankinson (2017)	Housing preferences	655	✓	803		
Bonikowski and Zhang (2017)	Populism	642	✓	421	Qualtrics	1,035
Kaufman (2018)	Survey bias	272	✓	524		
Kaufman, King and Komisarchik (2018)	District compactness	373	✓	764		
Saha annd Weeks (2018)	Candidate ambition	550			SSI	1200
Mozer et al. (2018)	Article similarity	226	✓	336		

*In the manuscript, we present a table summarizing the 6 replications we conducted explicitly for this study. Here, we present 10 additional replications reported by other researchers while using DLABSS. "Other" column indicates the first sample, of which we are aware, other than MTurk or DLABSS, on which the study was carried out and is not an exhaustive list of replications. The first N column is the number of subjects on DLABSS, the second N is the number of subjects on MTurk, and the third N is the number of subjects on different platforms, where applicable. A * next to the ✓for MTurk indicates that we carried out the MTurk replication ourselves.*

References

Bonikowski, Bart and Yueran Zhang. 2017. "Populism as dog-whistle politics: Anti-elite discourse and sentiments toward minorities in the 2016 presidential election." Working Paper, Harvard University.

Enos, Ryan D. 2017. *The space between us: Social geography and politics*. New York: Cambridge University Press.

Enos, Ryan D and Christopher Celaya. 2018. "The effect of segregation on intergroup relations." *Journal of Experimental Political Science* 5(1):26–38.

Enos, Ryan D. and Riley Carney. 2017. "Conservatism and fairness in contemporary politics: Unpacking the psychological underpinnings of modern racism." Working paper, Harvard University, `https://scholar.harvard.edu/files/rkcarney/files/carneyenos.pdf`.

Gadarian, Shana Kushner and Bethany Albertson. 2014. "Anxiety, immigration, and the search for information." *Political Psychology* 35(2):133–164.

Hainmueller, Jens and Michael J Hiscox. 2010. "Attitudes toward highly skilled and low-skilled immigration: Evidence from a survey experiment." *American Political Science Review* 104(01):61–84.

Hankinson, Michael. 2018. "When do renters behave like homeowners? High rent, price anxiety, and NIMBYism." *American Political Science Review* 112(3):473–493.

Kam, Cindy D and Elizabeth N Simas. 2010. "Risk orientations and policy frames." *The Journal of Politics* 72(02):381–396.

Kaufman, Aaron. 2018. "An automated method to estimate bias in survey questions." Working Paper, Harvard University. Draft available at `http://www.aaronrkaufman.com/paper/surveybias/`.

Kaufman, Aaron, Gary King and Mayya Komisarchik. Forthcoming. "How to measure legislative district compactness if you only know it when you see it." *American Journal of Political Science* .

Kaufman, Aaron and Matthew Kim. 2017. "Sequential blocked randomization for internet-based survey experiments." Working Paper, Harvard University.

Krosch, Amy R and David M Amodio. 2014. "Economic scarcity alters the perception of race." *Proceedings of the National Academy of Sciences* 111(25):9079–9084.

Krosch, Amy R, Leslie Berntsen, David M Amodio, John T Jost and Jay J Van Bavel. 2013. "On the ideology of hypodescent: Political conservatism predicts categorization of racially ambiguous faces as Black." *Journal of Experimental Social Psychology* 49(6):1196–1203.

Mahler, Daniel. 2016. "Do altruistic preferences matter for voting outcomes?" Working Paper, University of Copenhagnen. Draft available at: `https://editorialexpress.com/cgi-bin/conference/download.cgi?db_name=SCW2016&paper_id=126`.

Mozer, Reagan, Luke Miratrix, Aaron R. Kaufman and L. Jason Anastasopoulos. 2018. "Matching with text data: An experimental evaluation of methods for matching documents and of measuring match quality." Draft available at `https://arxiv.org/abs/1801.00644`.

Rasinski, Kenneth A. 1989. "The effect of question wording on public support for government spending." *Public Opinion Quarterly* 53(3):388–394.

Saha, Sparsha and Ana Catalano Weeks. 2018. "Ambitious women: Gender and voter perceptions of candidate ambition." Working paper, Harvard University.

Sears, David O. and Donald R. Kinder. 1971. Racial tensions and voting in Los Angeles. In *Los Angeles: viability and prospects for metropolitan leadership*, ed. Werner Z Hirsch. New York: Praeger.

Tomz, Michael. 2007. "Domestic audience costs in international relations: An experimental approach." *International Organization* 61(04):821–840.

Tversky, Amos and Daniel Kahneman. 1981. "The framing of decisions and the psychology of choice." *Science* 211(4481):453–458.
