# Supplementary material for: Online volunteer laboratories for human subjects research
Source: PLoS One. 2019 Aug 28;14(8):e0221676. doi: 10.1371/journal.pone.0221676 (PMC6713332; doi:10.1371/journal.pone.0221676)
Supplement: S4 Appendix — This appendix includes additional information on the design and results of individual response quality tests for volunteer and paid subjects. (PDF) [file pone.0221676.s004.pdf]

Appendices for *Online Volunteer Laboratories for Human Subjects Research*

S4 Appendix: Supplemental Materials for Study 4

This appendix includes additional information on the design and results of individual response quality tests for volunteer and paid subjects.

A Sample Composition

We provide an overview of the sample sizes for each of the tests of response quality discussed in the manuscript. Due to a combination of missingness in demographic covariates, plus some attrition throughout the survey, the sample size for each test ranges from around 1,400 to 1,700 when combined across survey topics and volunteer status (Table A). The table suggests even balance across paid and volunteer subjects in missingness and attrition.

Table A: Sample Sizes by Response Quality Test, Survey Topic, and Volunteer Status.

	Sample Size	By Survey Topic		By Volunteer Status	
	Combined	FEP	Secular	MTurk	Volunteer
Time answering open-ended 2	1,477	719	758	755	722
Time answering open-ended 1	1,411	664	747	732	679
Time reading prompt		750		806	741
Time answering short items		697		740	698
Straightlining	1,685	822	863	871	814
Open-ended Response Effort	1,466	708	758	755	711
Open-ended question 2 length	1,475	718	757	753	722
Open-ended question 1 length	1,484	725	759	761	723
Open-ended Response Quality	1,466	708	758	755	711
Commital question 2	1,494	730	764	771	723
Commital question 1	1,493	730	763	771	722
Consistency	1,499	734	765	773	726
Skipping	1,509	741	768	774	735
Attention Check			773	384	389

We compare the demographic balance across the paid and unpaid samples that took the

response quality surveys in Table B. We show the samples are balanced on many variables, but that the DLABSS sample tends to be older, richer, whiter, more religious, and more politically conservative. As noted in the body of the paper, many of these differences actually make DLABSS more similar to the larger US population and are the result of intentional targeting of certain populations in DLABSS recruitment efforts. Notably however, DLABSS does contain more missingness in demographic variables.

Table B: Demographics in DLABSS and MTurk.

	DLABSS	*MTurk*
Female	49.0% (1.8)<br>[5.1%]	50.3% (1.8)<br>[0.1%]
Education (mean years)	16.0 (0.1)<br>[3.7]	14.9 (0.1)<br>[0.0]
Age (mean years)	56.0 (0.6)<br>[2.4]	38.7 (0.5)<br>[0.0]
Mean income	$60,717 ($1,594)<br>[9.1]	$44,727 ($1,192)<br>[0.7]
Median income	$55,000	$37,500
Race		
White	88.9 (1.1)<br>[2.1]	77.0 (1.5)<br>[0.0]
Black	2.4 (0.5)	6.8 (0.9)
Hispanic	4.5 (0.7)	7.4 (0.9)
Attend Religious Service Weekly	33.6 (2.2)<br>[2.5]	15.0 (1.8)<br>[0.0]
Religion		
Protestant	45.7 (2.3)<br>[0.4]	34.4 (2.4)<br>[0.0]
Other Christian	4.9 (1.0)	2.1 (0.7)
Other	14.8 (1.6)	19.9 (2.0)
None	34.6 (2.2)	43.7 (2.5)
Party Identification		
Democrat	37.4 (1.7)<br>[7.9]	50.2 (1.8)<br>[3.2]
Independent	30.5 (1.6)	23.8 (1.5)
Republican	32.1 (1.7)	26.0 (1.6)
Liberal	54.4 (2.3)<br>[4.7]	60.5 (2.5)<br>[0.0]
Speak English at Home	99.8 (0.2)<br>[2.0]	100.0 (0.0)<br>[0.0]
N	476-843	387-782

Standard errors are in parentheses. Percent missing in brackets. N varies across questions due to missingness and the religious questions only appearing on one survey.

B Summary of Response Quality Tests

Table C provides a summary of each measure of response quality employed in this study.

Table C: Tests of Response Quality

Quality Dimension	Test	Measurement
Time Investment	Time answering open-ended items	Seconds spent on response page for each of two open-ended survey items (presented as an average and separately)
	Time reading prompt	Seconds viewing an article of about 400 words
	Time answering short items	Seconds spent on response page with five short-answer/multiple choice items pertaining to article
Straightlining	Straightlining in matrix-style question grids	Share of three bidirectional question matrices with entirely uniform answers
Open-Ended Investment	Subjective effort	Effort score out of five (with five being most perceived effort) given by two human coders for one open-ended survey item
	Response length	Number of characters in response to each of two open-ended survey items (presented as an average and separately)
	Subjective response quality	Summary of three dimensions coded 0 or 1 by two human coders for one open-ended survey item: whether response is long, topical, and complete
Committal Answers	"Don't Know" answers regarding commitment to action	Whether respondent answers "Don't know" to each of two committal questions (presented as an average and separately)
Consistency	Contradicting previous responses	Whether subject direction of subjects' response to a multiple-choice question contradicts previous response in grid-style question
Skipping	Skipping questions when given opportunity	Whether subject picks the "Skip" option in a multiple choice opinion question about policy opinions
Attention Check	Noticing embedded "attention check"	Whether subject answers a factual short-item question with "yes," rather than the true answer, as directed in a reading prompt

C Response Quality by Survey Topic

In the body of the paper, we present plots including the standardized coefficients for volunteer response quality across all tests, including demographic covariates such as age, income, education, race, frequency of religious service attendance, religious tradition, political ideology, and party identification and dummy variable for the survey topic. We report results using the combined survey data across studies. Here, we provide additional background information on test design and present coefficient plots separated by survey (Figure A). We also present the same coefficient plot for the bivariate regression of each quality test on volunteer status, without controlling for demographic covariates (Figure B). Due to the embedded attention check in the secularism version of the survey, we can only present results for time investment into reading the article and answering subsequent questions for the foreign economic policy survey. Likewise, results for the attention check are limited to the secularism survey.

Figure A: Standardized "Volunteer" Coefficients for All Response Quality Tests, by survey with covariates

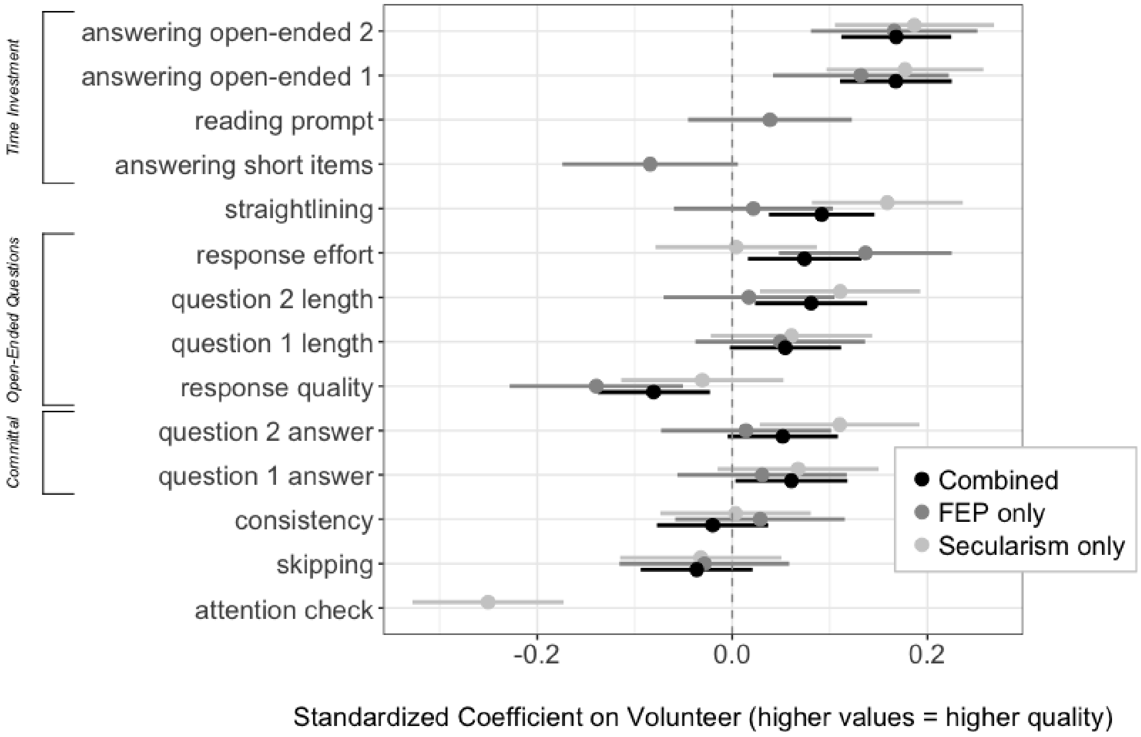

Figure B: Standardized "Volunteer" Coefficients for All Response Quality Tests, by survey without covariates

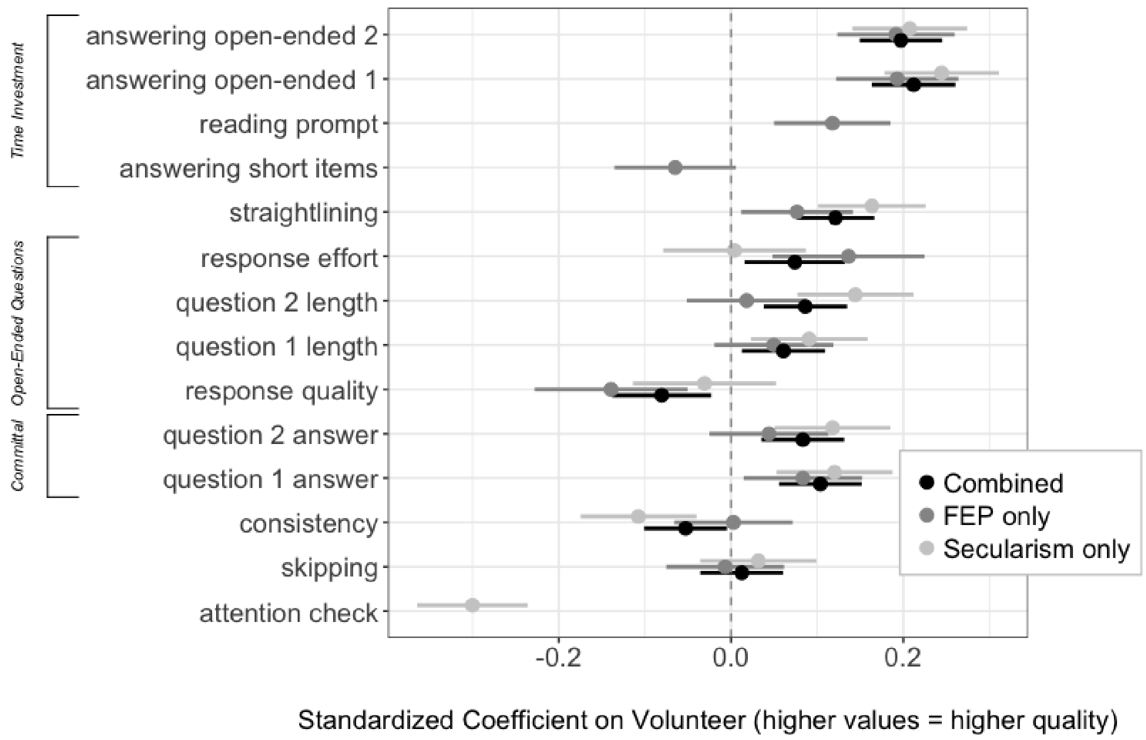

D Design and Results Details for All Tests

Time Investment: One measure of response quality is time investment in answering survey questions. In general, we consider longer time investment to be a reflection of more careful and higher quality responses. Our primary test involves a reading prompt followed by several short questions. We chose this format because reading prompts take more time than standard survey questions and subjects have no restrictions on how much time they need to spend reading the prompt. This test thus enables us to identify potential variation in time spent reading the prompt and answering questions, the latter of which are not available until a respondent confirms they have finished reading.

For the secularism questionnaire the prompt is an excerpt of a *New York Times* op-ed about the rise of secularism and atheism in American society. For the foreign economic policy study, respondents were invited to read an excerpt from *Foreign Policy Magazine* on Chinese global economic activities. The prompts are structurally similar in terms of their number and length of paragraphs, their emphasis on providing objective facts on the topic, and their overall length. Each prompt is about 400 words. While we include a time investment test in both surveys, we also embed an attention check in the secularism survey, invalidating comparisons across the two survey instruments for time investment. Thus, we present results only for the foreign economic policy survey.

For our primary test of subjects' investment of time into their survey responses, we measure the number of seconds respondents spend reading the article prompt before clicking "next" to answer questions about it. We also measure the number of seconds spent responding to five short-answer or multiple choice questions about the article. Two additional tests of time investment are the number of seconds respondents spend answering two open-ended questions later in the survey. For all of our time investment tests, the dependent variable is the number of seconds spent before clicking to the next page. Positive coefficients would

mean volunteers spend more time on the task, and we interpret more seconds spent as an indication of higher response quality. For this analysis, we trim the outer 5th percentile of time to eliminate extreme outliers.

Both with and without controls, volunteers spend more time than paid subjects on the reading prompts, though the difference becomes statistically insignificant with controls. For a random half of subjects, we also included a reminder to respondents to take their time reading, as they would not be permitted to click "back" to view the article. We do not analyze that effect here. Volunteers spent slightly less time responding to the block of questions about the article they had read. Volunteers spent more time responding to open-ended questions on both surveys than paid subjects, controlling for subject characteristics.

Straightlining: We examine whether paid and volunteer subjects have significantly different propensities to engage in straightlining. Straightlining is a well-known phenomenon in survey research in which respondents rush through a survey and provide the same response for many questions without actually reading and considering the question content. More complex forms of straightlining include patterned or random responses, which are considerably more difficult for investigators to detect. With well-designed survey questions that naturally induce variation, less straightlining signals a higher quality response.

We design a test for straightlining that presents respondents with several matrix-style question blocks. This type of questioning is arguably especially vulnerable to straightlining. This is because several grid-style questions are presented on a single page, and answer choices to each question are located in close proximity to each other. Figure C offers an example of a question block from the foreign economic policy survey. We include seven of these blocks in each survey.

Question blocks within each survey vary in terms of their directionality, that is, the extent to which choosing the same answer for each question would reflect consistent, logical attitudes. Including bidirectionality in some of these question blocks allows us to detect

Figure C: Sample Question Matrix used in Straightlining Tests

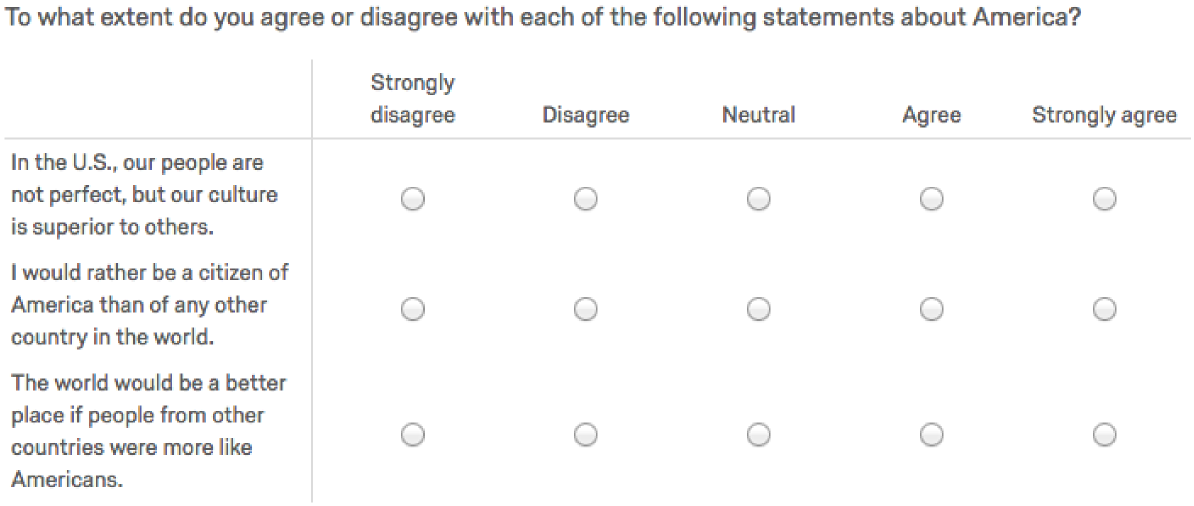
To what extent do you agree or disagree with each of the following statements about America?

	Strongly disagree	Disagree	Neutral	Agree	Strongly agree
In the U.S., our people are not perfect, but our culture is superior to others.	○	○	○	○	○
I would rather be a citizen of America than of any other country in the world.	○	○	○	○	○
The world would be a better place if people from other countries were more like Americans.	○	○	○	○	○

straightlining in instances where a respondent with consistent preferences should not choose the same answer category. For example, in one of the question blocks in the foreign economic policy survey, we ask respondents whether they thought both “border walls” and “more open borders” are beneficial for American interests. In this case, vertical straightlining behavior, in which a respondent chooses the same preference for every question, would be strong evidence that a subject is rushing through a survey. While respondents see multiple blocks, some with bidirectional response items and others with unidirectional response items, our measure of straightlining is constructed including only those blocks with bidirectional responses, i.e., those on which a respondent paying attention would *not* be expected to straightline naturally.

Arguably the most straight-forward test of straightlining is to simply create a variable that measures whether a subject engaged in vertical straightlining—meaning he or she answered the same response category for every question—for each question block. This type of answer behavior, if detected, is perhaps the most egregious form of straightlining and thus

represents a useful first step. Thus, we use a simple binary operationalization of whether a respondent engaged in vertical straightlining by offering a uniform response category for every item within a straightlining block. Since each of our surveys had three total bidirectional grid-style question matrices, our dependent variable in this test is the proportion of these three questions in which the subject did offer a single uniform response category.

As depicted in Figure B, the volunteer coefficient for both the economic policy and secularism surveys is statistically distinguishable from the null in the bivariate regressions, in the direction of a higher quality response. In a multivariate setup (Figure A), volunteers were less likely to engage in straightlining in the secularism survey than paid respondents, but there was no significant difference between volunteer and paid respondents in the survey related to economic policy.

Open-Ended Investment: We next test the possibility that subjects motivated by different incentives vary in the quality of their open-ended survey answers. We include multiple open-ended response questions in each survey, which provide subjects with the opportunity to expand on their other answers, leave feedback for the research team, or otherwise write additional content. Researchers relying on open-ended response data may perceive higher quality responses as those which are longer and include more interesting content. Researchers might also value open-ended responses that provide suggestions for improving the study.

In the body of the manuscript and Figure A, we present results on open-ended response quality based on a human-coded, subjective, composite quality score with several dimensions. Two undergraduate students coded each open-ended response. They provided a subjective 1-5 ranking of the amount of effort they perceived the subjects invested in the item. They also provide binary 0-1 score for whether each response was long, topical, and complete. These last three dimensions of quality are aggregated into a subjective quality score. One of the authors also coded all responses on which the two coders had a difference of more than

2 points in the 5-point effort scale or a disagreement on the binary score for any of the three quality dimensions. For both effort and the composite quality score, higher values represent higher quality responses.

We also test open-ended response quality by checking whether respondents significantly differ in the number of characters written in response to open-answer prompts. We argue that increases in this measure on average indicate a higher quality response or, at least, greater participant investment in the survey.

For all open-ended tests, we differentiate between open-ended questions left blank due to skipping (in which a subject viewed an open-ended question but did not write anything) and those left blank because the subject had attrited prior to viewing the open-ended question. We include skipped questions as zeros in our analysis, but exclude attrited respondents from the analysis.

Figure A provides mixed quantitative support for the idea that respondent motivation impacts responses to open-ended questions. On both open-ended questions, volunteers wrote more characters than paid respondents for the secularism survey, though not the foreign policy survey. For the human-coded scores, on average across the surveys, volunteers scored higher on effort, but lower on response quality overall.

Non-committal responses: Our surveys also include tests of commitment. In this context, commitment refers to subjects' willingness to signal intensity of attitudes in their reported responses by stating their intent to support (or oppose) a cause with behavior beyond simply reported survey responses. We employ multiple measures of commitment in each survey. For example, in the secularization survey we ask whether respondents are willing to 1) sign a petition and 2) confront an individual about inappropriate conduct. Subjects can report their intent to partake or abstain from either behavior, or can choose a less committal answer such as "It depends" or "Unsure."

For our tests of commitment, we code a noncommittal answer as one in which a respon-

dent does not express intent to participate or abstain from participation. Choosing "unsure" or "it depends" was taken as a lower-quality response, though we address ambiguity about interpreting these results in our discussion of findings below.

Results on these items were mixed. In the absence of demographic covariates, volunteers were somewhat less likely to choose the noncommittal response for all but the second committal item on the economic policy survey. However, after the inclusion of demographic covariates, these distinctions are no longer significant in many cases.

We note that a response of "unsure" can be interpreted in various ways with respect to response quality. Although one interpretation of quality is to expect higher-quality responses to include fewer non-committal responses, alternative interpretations are possible. For example, more unsure responses might be an expression that respondents are less likely to engage in cheap talk. Future research could work to examine this distinction in greater detail.

Inconsistency: We examine consistency across a subject's answers within a survey. In general we perceive higher consistency as a measure of higher response quality: if respondents report unstable or illogical opinions, preferences, or other responses in a short study, there is certainly reason to doubt whether such responses reflect actual attitudes (Achen 1975). Inconsistency alternatively may simply be a proxy for lower levels of attention paid by subjects to the study content, an equally worrisome possibility.

To explore the possibility that paid and unpaid subjects differ in their cognitive invested in the content of a study, we design a test that asks subjects the same question twice. The first version of the question is embedded in one of the straightlining blocks discussed above. The second version is a the same question phrased differently and presented in a different format, in this case as a standalone multiple choice question later in the survey. We randomize whether or not a respondent is first reminded that he or she has already been asked about this issue. We do not analyze that effect here.

We score this metric by creating a variable that measures whether there is directional consistency across the two questions, that is, if a respondent's reported preferences are in the same direction, if not to the same degree. To require exact equality in both direction and degree seemed to be a test so stringent that it was inconsistent with what the literature would anticipate as reasonably high-quality and consistent (Ansolabehere, Rodden, and Snyder 2008).

Figure A depicts the likelihood of volunteer versus paid subjects responding in a consistent direction on the two items. More consistency is seen as an indicator of higher-quality responses. For the most part, paid and unpaid subjects did not statistically differ on these items. As one exception, volunteer survey responses were arguably of lower quality in relation to response consistency on the secularism version of the survey in the bivariate framework. However, this distinction did not hold once control variables were introduced.

Skipping: We design a simple test to detect a subject's propensity to skip questions. Because question skipping creates missing data, which can create bias if not corrected (King et al. 2001), subjects who skip fewer questions are typically more desirable than those who skip more. We design this test by creating a question about individual's preferences for a certain policy. For example, in the secularization survey we ask a question related to churches' rights to engage in political activities, and for the foreign economic policy survey we ask about whether the government should encourage more free trade. For these questions respondents could choose, "I'm not sure. Skip." as an answer choice beyond the standard support-oppose scale. To measure skipping, we simply create a variable that receives the value of "1" if a respondent chose to skip the question.

It is worth noting that the choice to skip in survey questions can be interpreted in multiple directions in relation to response quality. On the one hand, skipping may represent taking an "easy way out," wherein subjects avoid engaging with a cognitively challenging question or are simply rushing to finish and, thus, may represent low quality responses. On

the other hand, skipping may genuinely represent uncertainty among subjects and may be more desirable than other response strategies, such as choosing an answer at random. For subjects who are aware that they are uninformed on a particular policy issue, skipping may represent a reasonable, high-quality choice. There are no significant differences between paid and unpaid respondents in this test of skipping.

Attention Check: We also embed an attention check, or "screener" in our secularism survey instrument. As noted above, we task subjects with reading an article and responding to questions about that article. In order to ensure respondents were actually reading the article, not merely opening the prompt and then doing something else before proceeding, we included a sentence in the middle of the article that asked respondents to reply "yes" to an open-ended question in the question block following the article, rather than answering the question. We include this test only in the text of the article in the secularism survey.

In the manuscript, we discuss ways in which volunteer subjects may potentially exhibit less propensity for attrition than paid subjects. Figure D displays the mean and median attrition rates and completion times for DLABSS studies.

E Attrition and Response Time

Figure D shows the frequency of attrition rates by survey and the average time to complete by survey across 120 studies hosted on DLABSS.

Figure D: DLABSS Study Attrition Rates and Completion Times

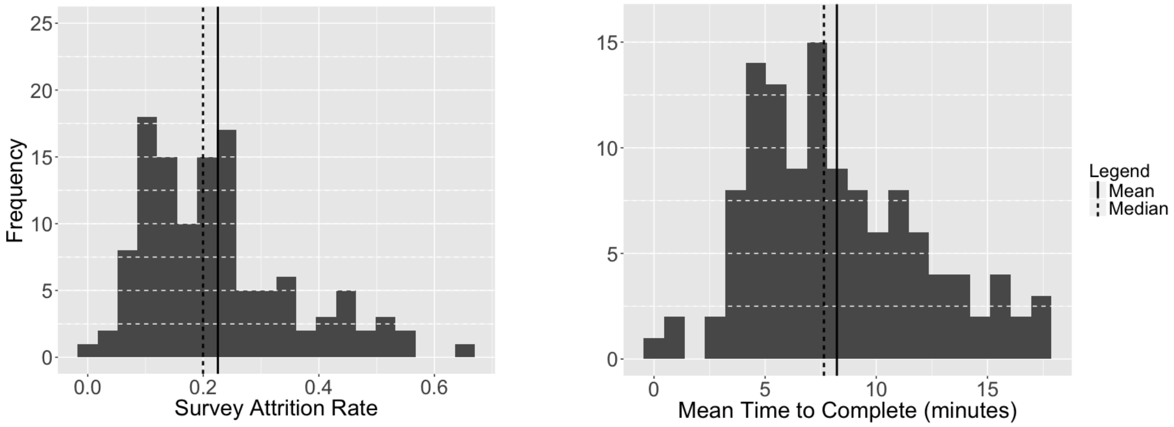

Left figure is total attrition by study and right figure is mean time to complete by study for 120 studies hosted by DLABSS.

References

Achen, Christopher H. 1975. "Mass political attitudes and the survey response." *American Political Science Review* 69(04): 1218–1231.

Ansolabehere, Stephen, Jonathan Rodden, and James M Snyder. 2008. "The strength of issues: Using multiple measures to gauge preference stability, ideological constraint, and issue voting." *American Political Science Review* 102(2): 215–232.

King, Gary, James Honaker, Anne Joseph, and Kenneth Scheve. 2001. "Analyzing incomplete political science data: An alternative algorithm for multiple imputation." *American Political Science Review* 95(1): 49–69.
